# Effect of host genetics and gut microbiome on fat deposition traits in pigs

**DOI:** 10.3389/fmicb.2022.925200

**Published:** 2022-09-20

**Authors:** Yuan Wang, Ping Zhou, Xiang Zhou, Ming Fu, Tengfei Wang, Zuhong Liu, Xiaolei Liu, Zhiquan Wang, Bang Liu

**Affiliations:** ^1^Key Laboratory of Agricultural Animal Genetics, Breeding, and Reproduction of Ministry of Education, and Key Laboratory of Swine Genetics and Breeding of Ministry of Agriculture, Huazhong Agricultural University, Wuhan, China; ^2^The Cooperative Innovation Center for Sustainable Pig Production, Wuhan, China; ^3^The Engineering Technology Research Center of Hubei Province Local Pig Breed Improvement, Wuhan, China; ^4^Hubei Hongshan Laboratory, Wuhan, China; ^5^Livestock Gentec, Department of Agricultural, Food and Nutritional Science, University of Alberta, Edmonton, AB, Canada

**Keywords:** percentage of leaf fat, intramuscular fat content, heritability, microbiability, microbe

## Abstract

Fat deposition affects meat quality, flavor, and production in pigs. Fat deposition is influenced by both genetics and environment. Symbiotic microbe with the host is an important environmental factor to influence fat deposition. In this study, the fat deposition traits were measured in 239 individuals obtained from Tongcheng pigs × Large White pigs resource population. The interactions between genetics and gut microbiome in fat deposition traits were investigated through whole-genome sequencing and cecum microbial 16S ribosomal RNA sequencing. The results showed that the percentage of leaf fat (PL) and intramuscular fat content (IMF) were significantly influenced by host genetics–gut microbiome interaction. The effects of interactions between host genetics and gut microbiome on PL and IMF were 0.13 and 0.29, respectively. The heritability of PL and IMF was estimated as 0.71 and 0.89, respectively. The microbiability of PL and IMF was 0.20 and 0.26, respectively. Microbiome-wide association analysis (MWAS) revealed *Anaeroplasma*, *Paraprevotella*, *Pasteurella*, and *Streptococcus* were significantly associated with PL, and *Sharpea* and *Helicobacter* exhibited significant association with IMF (*p* < 0.05). Furthermore, *Paraprevotella* was also identified as a critical microbe affecting PL based on the divergent Wilcoxon rank-sum test. Overall, this study reveals the effect of host genetics and gut microbiome on pig fat deposition traits and provides a new perspective on the genetic improvement of pig fat deposition traits.

## Introduction

Fat deposition is closely related to carcass and meat quality traits in pigs. Fat deposition in different parts of the body has different effects. Subcutaneous backfat thickness is an important target trait in pig breeding. There is a negative correlation between backfat thickness and carcass lean percentage. In the viscera, fat could support and protect organs, and it affects dressing percentage and feed consumption in pigs. In addition, intramuscular fat content (IMF) is an important factor determining the tenderness, juiciness, flavor, meat quality characteristics, and consumer acceptance (Gao and Zhao, 2009). IMF is positively correlated with juiciness and flavor of meat (Fortin et al., 2005). Thus, fat deposition is an important economic trait of pigs, which affects meat quality and production in pig. Furthermore, the physiological traits and biochemical indices of pigs are similar to humans, thus, pig can be used as a model animal for studying human obesity (Hishikawa et al., 2005).

Fat deposition traits are a type of quantitative traits with moderate-to-high heritability. This quantitative trait is influenced by heredity and environment. The gut microbiota that are symbiotic with the host are irreplaceable environmental factors. The gut microbiome is known as the second genome of humans (Zhu et al., 2010), which inhabits 10 times more gut microbial cells than human cells (Savage, 1977). Pig has richer gut microbes and is considered to be the main model animal of human obesity and disease research (Hishikawa et al., 2005; Merlotti et al., 2017). A reference gene catalog of pig gut microbiome with a total of 7.69 million genes has been established by metagenome sequencing of 278 pig fecal microbiome (Xiao et al., 2016).

The influence of gut microbe on host phenotype, the ratio of microbial relative abundance variance to the phenotype variance, is defined as “microbiability” (Difford et al., 2018). Microbiability has been used for the studies in pigs (Camarinha-Silva et al., 2017; Tang et al., 2020), cattle (Difford et al., 2018), and chicken (Wen et al., 2019b). According to the microbiability of traits, cecal microbiota has a greater contribution to fat deposition than other large intestine microorganisms (Wen et al., 2019b; Tang et al., 2020). Moreover, the *Treponema* in cecum has been reported to be associated with feed conversion (Quan et al., 2018). The *Prevotellaceae ucg-001* in cecum is positively correlated with backfat thickness and IMF (Tang et al., 2020). Besides, the cecum exhibits high capabilities of degradation and digestion, whose metabolites can provide energy for the host (Yang et al., 2016). Therefore, the cecum could be considered as the representative of gut segments for fat deposition research.

However, the effect of interactions between host genetics and gut microbiome on fat deposition traits remains largely unclear. In our study, the resource population was obtained by crossing a Chinese local breed (Tongcheng pigs) and a commercial breed (Large White pigs). We measured fat deposition traits (backfat thickness at shoulder (SBFT), loin (LBFT), rump (RBFT), average backfat thickness (ABFT), percentage of leaf and caul fat (PL, PC, and PLC), and intramuscular fat content (IMF)). We performed whole-genome sequencing and cecal microbial 16S ribosomal RNA sequencing of 239 pigs to determine whether there are any interactions between the host genetics and gut microbiome. Then, we estimated heritability and microbiability for the detected interactions between host genetic–gut microbiomes and further screened candidate microbes influencing traits. This study provides a new perspective and opportunity for the genetic improvement of pig fat deposition traits.

## Materials and methods

### Resource population, fat deposition traits measured, and sample collection

The resource population is an advanced generation intercross population. F1 was obtained by crossing 36 Tongcheng sows (a Chinese local breed) with 11 Large White boars (a commercial breed). The F1 sows and boars were subsequently intercrossed to produce F2. Then, intercross was performed in each generation until F9 population was produced. In this study, a total of 239 individuals from 9 to 10 intercross generations including 119 castrated boars and 120 castrated sows were used for further analysis. All the pigs were provided by Yunzhi Farm (Tongcheng Country, Hubei province, China) with coincident feeding and management conditions. All individuals were healthy, and they were not administered any antibiotics before being slaughtered.

The phenotypes of 239 pigs were collected, including live body weight (BW) before slaughter, carcass weight (CW), backfat thickness at the thickest shoulder (SBFT), loin (LBFT), rump (RBFT), weight of leaf fat, weight of caul fat, and intramuscular fat content (IMF). Backfat thickness was measured using Vernier caliper. The average backfat thickness (ABFT) was an average of three measurements of backfat thickness at the shoulder, loin, and rump. The weight of leaf and caul fat was measured using a scale with an accuracy of 0.01 kg. The percentage of leaf fat (PL) was calculated as the weight of leaf fat divided by CW. Similarly, percentage of caul fat (PC) was calculated as the weight of caul fat divided by CW. The percentage of leaf and caul fat (PLC) was sum of PL and PC. A near-infrared spectroscopy analyzer was used to measure the IMF of the longissimus dorsi samples. The longissimus dorsi samples were collected from the penultimate 3rd to 4th thoracic vertebrae, about 20–30 cm long. The gut contents of 239 individuals were sampled from cecum after pigs were slaughtered, snap-frozen in liquid nitrogen, and stored at −80°C until sequencing. Spleen tissues were collected into 50 ml centrifuge tubes containing 75% alcohol by volume and stored at −20°C.

All the animal experiments in this study were approved by the Animal Care and Use Committee of Huazhong Agricultural University.

### Whole-genome sequencing and analysis

Genomic DNA samples were extracted from spleen of 239 pigs to construct the whole-genome sequencing libraries. Each library was sequenced using 150 bp paired-end reads with a HiSeq X5 instrument (Illumina). To minimize mapping error, low-quality reads were removed using FastQC software. The clean reads from each pig were aligned to the porcine reference genome using the Burrows-Wheeler Alignment tool (BWA ver 0.7.15) with default parameters. We subsequently used the Picard toolkit to sort the alignment results and remove potential PCR duplicate reads. The resultant alignments were indexed using SAMtools (ver 1.6) and processed with the Genome Analysis Toolkit (GATK, ver 3.7). To call variants, we set a minimum quality score as 20 based on the bases and mapped reads. The single nucleotide polymorphisms (SNPs) of each pig were combined to obtain a common dataset of single nucleotide polymorphism (SNP) data, and the dataset was processed by GATK. Finally, the software PLINK (ver 1.90) was used for quality control of the obtained dataset with the following filtering criteria: SNP call rate >80%, minor allele frequency >1%, and Hardy–Weinberg equilibrium *p*-value <10^–6^.

### 16S ribosomal ribonucleic acid sequencing and analysis

First, the total DNA of cecal contents was extracted to construct sequencing library, and 30 ng genomic DNA samples and their corresponding fusion primers were used to prepare PCR reaction system. PCR reaction was performed to amplify V3-V4 region of 16S ribosomal RNA (rRNA) with the primers of 338R (ACTCCTACGGGAGGCAGCAG) and 806R (GGACTACHVGGGGTWTCTAAT). A 468 bp segment was amplified by PCR with the number of tags of 50,000. The PCR products were purified by using Agencourt AMPure XP magnetic beads and dissolved in the Elution Buffer. Agilent 2100 Bioanalyzer was used to detect the fragment range and concentration of the library. According to the size of the inserted fragment, HiSeq platform was selected for pair-end sequencing. FLASH (Fast Length Adjustment of Short reads, ver 1.2.11) was used to splice the sequences. Based on the sequence overlapping relationship, the paired reads obtained from pair-end sequencing were assembled into tags. The assembled tags were clustered into operational taxonomic units (OTUs) using software USEARCH (ver 7.0.1090). Clustering analysis was performed by using UPARSE under 97% similarity to obtain representative sequence of each OTU. Afterward, OTU representative sequences were aligned against the Greengene database by ribosomal database project (RDP) classifier (ver 2.2) software to obtain the annotation at the levels of phylum, class, order, family, and genus.

### Analysis of interactions between host genetics and gut microbiome

#### Construction of genomic relationship matrix

A total of 14,139,625 filtered SNPs were used to estimate the genomic relationship matrix (GRM) using the HIBLUP software^1^ according to the following equation:

$$G\,={\mathrm{MM}}^{{}^{\prime }}/2\,\sum_{i\,=1}^{n}p_{i}\,\left(1-p_{i}\right)$$

In which M indicates m (the number of individuals) × n (the number of loci) matrix, and *p*_*i*_ is the frequency of the coded allele.

#### Construction of microbial relationship matrix

All the OTUs were used to construct the microbial relationship matrix (MRM) by R script (Wen et al., 2019b) based on the following equation:

$$m_{\mathrm{ij}}=\frac{1}{N}\,\sum_{o\,=1}^{N}\frac{\left(X_{\mathrm{io}}-X_{o}\right)\,\left(X_{\mathrm{jo}}-X_{o}\right)}{\sigma_{o}^{2}}$$

In which *m*_*ij*_ is the estimation of microbial similarity in cecum of individual i and individual j; *X*_*io*_ and *X*_*jo*_ indicate the relative abundance of OTU o in the cecum of individual i and individual j; *X*_*o*_ represents the average relative abundance of OTU o in cecum of the whole population; $\sigma_{o}^{2}$ is the variance of OTU o relative abundance; and N is the total OTU count in cecum.

#### Interactions between host genetics and gut microbiome

The following multiple random effects model was established to estimate variance components of the target traits using HIBLUP software (see text footnote 1).

$$y=1\,\mu +Z_{1}\,g+Z_{2}\,m+Z_{3}\,a+e$$

In which *y* is the n × 1 vector of the fat deposition traits; μ is the overall mean; *g ∼ N (0*,$G\,\sigma_{g}^{2}$) is the q × 1 vector of host genetic random effect, where *G* and $\sigma_{g}^{2}$ are the GRM and host genetic variance; *m ∼ N (0*,$M\,\sigma_{m}^{2}$) is the q × 1 vector of gut microbiome random effect, where *M* and $\sigma_{m}^{2}$ are the MRM and gut microbiome variance; *a ∼ N (0*,$A\,\sigma_{a}^{2}$) is the q × 1 vector of interactions between host genetics and gut microbiome random effect, where *A* and $\sigma_{a}^{2}$ are the GRM × MRM and variance of interactions between host genetics and gut microbiome; *e ∼ N (0*,$\sigma_{e}^{2}$)is an n × 1 vector of residual effect, where $\sigma_{e}^{2}$ is the residual variance; *Z*_1_, *Z*_2_, and *Z*_3_ are, respectively, the corresponding incidence matrices of *g*, *m*, and *a*.

### Estimation of heritability of fat deposition traits

A total of 14,139,625 filtered SNPs were used to estimate the principal components and heritability based on GRM using genome-wide complex trait analysis (GCTA) software [ver 1.93.1; (Yang et al., 2011)] according to the following equation:

$$y=K\,c+g_{1}+\varepsilon$$

where *y* is a vector of the phenotype; *c* is a vector of fixed covariates, including sex effect and body weight (BW); *K* is the matrix corresponding to *c*; and *g*_*1*_ is a vector of the total effects of all SNPs following *g*_*1*_ *∼ N (0*,$G^{\prime }\,\sigma_{g_{1}}^{2}$) where *G*′and $\sigma_{g_{1}}^{2}$ are the GRM and genetic variance, respectively; and ε is the residual effect. The *G*′ estimation equation is as follows:


$$g_{\mathrm{jk}}=\frac{1}{N}\,\sum_{i\,=1}^{N}\frac{\left(X_{\mathrm{ij}}-2\,p_{i}\right)\,\left(X_{\mathrm{ik}}-2\,p_{i}\right)}{2\,p_{i}\,\left(1-p_{i}\right)}$$

In which *g*_*jk*_ is the genetic similarity between individual j and individual k; *X*_*ij*_ and *X*_*ik*_ represent the number of reference alleles in individual j and individual k; *p*_*i*_ is the reference allele frequency; and N is the SNP number.

### Estimation of microbiability of fat deposition traits

The microbiability is referred to the ratio of cecum microbial variance to phenotypic variance, and it was calculated by the following equation:

$$y=K\,c+m_{1}+\varepsilon$$

where *y* and ε are phenotype and residual effect vectors, respectively. *m*_*1*_ is the gut microbiota effect following the *m*_*1*_ *∼ N (0, M*$\sigma_{m_{1}}^{2}$*).* The microbiability was estimated by GCTA software with the MRM substituted for GRM. In our study, ***c*** contained six covariates, namely, covariates 1–6. Covariate 1 included BW and the first five host genetic principal components (PCs); covariate 2 included BW and the first two PCs generated at SNPs which were significantly associated with PL and IMF; covariate 3 consisted of BW, the first five host genetic PCs, and the first two PCs generated at SNPs which were significantly associated with PL and IMF; covariate 4 included BW, sex, and the first five host genetic PCs; covariate 5 included BW, sex, and the first two PCs generated at SNPs which were significantly associated with PL and IMF; covariate 6 consisted of BW, sex, the first five host genetic PCs, and the first two PCs generated at SNPs which were significantly associated with PL and IMF. The first five PCs were generated by the genome relationship matrix to account for population structure.

The Bayesian information criterion (BIC) was calculated to select the model with optimal covariates. The smaller the BIC value was, the higher the model’s fitting level was. The BIC was calculated according to the following formula:

BIC=kIn⁢(n)-2⁢In⁢(L)

where *k* is the number of model parameters, *n* is the number of samples, and *L* is the likelihood value.

### Identification of candidate microbes with fat deposition traits

The divergent Wilcoxon rank-sum test and microbiome-wide association analysis (MWAS) were used to identify the candidate microbes for target traits.

#### Differences in genera, percentage of leaf fat, and intramuscular fat content between divergent groups

According to the distribution of fat deposition traits of the 239 individuals, the highest 20% (*n* = 48) and lowest 20% (*n* = 48) were formed into two extreme divergent groups. The Wilcoxon rank-sum test was used to test the significance in 56 genera with average relative abundance greater than 0.01% between the two groups. By the same method, microbes were grouped in terms of the relative abundance of the genus, and the significance in individual traits between the two divergent groups was tested by the Wilcoxon rank-sum test with the *P* < 0.05 as the chosen significant test criteria.

#### Microbiome-wide association analysis of percentage of leaf fat and intramuscular fat content

The MWAS between the fat deposition traits of 239 individuals and 56 genera whose average relative abundance was more than 0.01% was conducted using Gaston package^2^ in R software with the following equation:

y⁢=X⁢β+u+ε

*y* is the vector of fat deposition traits; β is a fixed effect vector, including 56 genera with average relative abundance value >0.01%; *u* is a random effect vector. At the same time, sex and BW are considered as covariates. *F*-test is used to determine the significance of the regression coefficients.

## Results

### Characterization of fat deposition traits and sequencing outcomes

Host fat deposition phenotype characteristics are presented in Table 1. There are three categories of fat depositions including subcutaneous fat (SBFT, RBFT, LBFT, and ABFT), viscera fat (PL, PC, and PLC), and intramuscular fat (IMF). All measured traits displayed a high coefficient of variation (15.39 to 42.69%) (Table 1). The distribution of each trait is shown in the Supplementary Figure 1. The host genetics was analyzed by the whole-genome sequencing, and the whole-genome sequencing information of the 239 individuals was analyzed subsequently. The SNPs were quality-controlled by Plink software. After quality control, a total of 14,139,625 SNPs was obtained for subsequent analysis.

**TABLE 1:** Summary of fat deposition traits in the resource population.

Trait	Sex	N	Mean	SD	CV (%)
SBFT (mm)	♂	119	52.09	8.01	15.39
	♀	120	52.57	8.54	16.24
	Total	239	52.33	8.27	15.8
LBFT (mm)	♂	119	27.88	6.83	24.49
	♀	120	29.19	7.16	24.54
	Total	239	28.54	7.01	24.58
RBFT (mm)	♂	119	29.06	6.49	22.32
	♀	120	32	7.51	23.46
	Total	239	30.53	7.16	23.44
ABFT (mm)	♂	119	36.34	6.4	17.61
	♀	120	37.92	7.01	18.49
	Total	239	37.13	6.75	18.16
PL (%)	♂	119	6.57	1.37	20.79
	♀	120	6.52	1.32	20.31
	Total	239	6.54	1.34	20.52
PC (%)	♂	119	3.52	0.94	26.66
	♀	120	3.64	0.97	26.58
	Total	239	3.58	0.95	26.62
PLC (%)	♂	119	10.08	2.05	20.37
	♀	120	10.16	1.98	19.52
	Total	239	10.12	2.02	19.91
IMF (%)	♂	119	2.13	0.91	42.69
	♀	120	1.89	0.64	34.12
	Total	239	2.01	0.8	39.6

SBFT, backfat thickness at shoulder; LBFT, backfat thickness at loin; RBFT, backfat thickness at rump; ABFT, average backfat thickness; PL, percentage of leaf fat; PC, percentage of caul fat; PLC, percentage of leaf fat and caul; IMF, intramuscular fat content; SD, standard deviation; CV, coefficient of variation.

**FIGURE 1:**
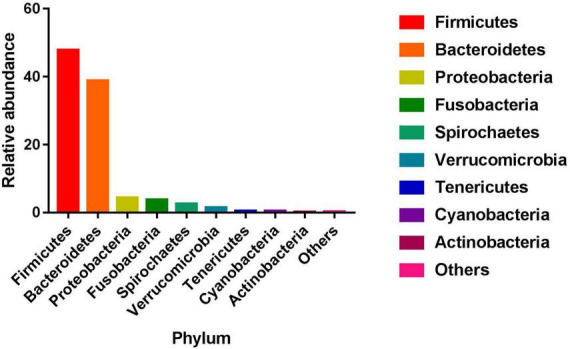
Phylum of relative abundance.

The gut microbiome was analyzed by the cecal 16S rRNA sequencing. The 16S rRNA sequencing analysis produced a total of 31,801,522 reads from the 239 samples with an average of 133,061 reads, and 2,324 OTUs were then clustered with 97% sequencing identity. Subsequently, these 2,324 OTUs were clustered into 19 phyla, 33 classes, 56 orders, 87 families, and 144 genera (Supplementary Table 1). At the phylum level, the phyla with relative abundance at the top five were *Firmicutes*, *Bacteroidetes*, *Proteobacteria*, *Fusobacteria*, and *Spirochaetes* (Figure 1). At the genus level, the average relative abundance of 56 genera was greater than 0.01% (Supplementary Table 2). The average Sobs index, Chao index, Ace index, Shannon index, and Simpson index of the microbiota are 782.92, 970.75, 962.85, 4.63, and 0.04, respectively (Supplementary Table 3).

### Influence of host genetic–gut microbiome interactions on percentage of leaf fat and intramuscular fat content

The effects of host genetics and gut microbiome on all traits were different (Table 2). The results indicated that host genetics (***g***) and gut microbiome (***m***) exhibited 0.62 and 0.20 independent effect on SBFT, 0.68 and 0.18 independent effect on LBFT, and 0.86 and 0.11 independent effect on RBFT, respectively. In addition, ABFT displayed 0.75 ***g*** effect and 0.18 ***m*** effect. Generally, the ***g*** effect was higher than the ***m*** effect in the presence of other environmental factors. RBFT exhibited the highest ***g*** effect among backfat thickness at three positions. The PC exhibited 0.40 ***g*** effect and 0.24 ***m*** effect. However, PL displayed 0.77 ***g*** effect, 0.10 ***m*** effect, and 0.13 effect of interactions between host genetics and gut microbiome (***a***). The multiple variance components completely explained the phenotypic variation of PL and IMF. The IMF showed 0.71 independent ***g*** effect and 0.29 ***a*** effect, but no independent ***m*** effect. The ***a*** effect of PL and IMF was estimated as 0.13 and 0.29, indicating that host genetic–gut microbiome interactions affected the formation of PL and IMF.

**TABLE 2:** Host genetics and gut microbiome effects for fat deposition traits.

Trait	*g*			*m*			*a*		
	$\sigma_{g}^{2}$	$\sigma_{g}^{2}/\sigma_{p}^{2}$	*P-value*	$\sigma_{m}^{2}$	$\sigma_{m}^{2}/\sigma_{p}^{2}$	*P-value*	$\sigma_{a}^{2}$	$\sigma_{a}^{2}/\sigma_{p}^{2}$	*P-value*
SBFT	65.92	0.62	0.23	20.92	0.20	0.14	0.00	0.00	0.25
LBFT	39.45	0.68	0.20	10.74	0.18	0.12	0.00	0.00	0.19
RBFT	66.19	0.86	0.22	8.07	0.11	0.10	0.00	0.00	0.25
ABFT	52.40	0.75	0.23	12.30	0.18	0.13	0.00	0.00	0.24
PL	1.54	0.77	0.18	0.19	0.10	0.09	0.26	0.13	0.18
PC	0.34	0.40	0.15	0.20	0.24	0.14	0.00	0.00	0.27
PLC	3.44	0.89	0.16	0.45	0.11	0.09	0.00	0.00	0.16
IMF	0.98	0.71	0.23	0.00	0.00	0.09	0.34	0.29	0.26

**g**, the effect of host genetics; **m**, the effect of gut microbiome; **a**, the effect of interactions between host genetics and gut microbiome; SBFT, backfat thickness at shoulder; LBFT, backfat thickness at loin; RBFT, backfat thickness at rump; ABFT, average backfat thickness; PL, percentage of leaf fat; PC, percentage of caul fat; PLC, percentage of leaf fat and caul; IMF, intramuscular fat content. The significant P-value bounds are P < 0.05.

### Heritability and microbiability of percentage of leaf fat and intramuscular fat content

The heritability of PL was estimated as 0.71 and that of IMF as 0.89. PL and IMF were mainly affected by host genetics in the resource population (Table 3). To estimate microbiability of PL and IMF, six models were established based on host genetics, population structure, sex, and BW (Supplementary Table 4). The BIC value was used to select optimal model fitting PL and IMF. The optimal model to calculate PL microbiability contained three covariates including BW, the first five host genetic principal components (PCs), and the first two PCs generated at SNPs significantly associated with PL. The microbiability of PL was estimated as 0.20 (Table 3). The optimal model to calculate IMF microbiability contained two covariates including BW and the first two PCs generated at SNPs significantly associated with IMF. The microbiability of IMF was estimated to be 0.26 (Table 3).

**TABLE 3:** Heritability and microbiability of PL and IMF in the resource population.

Trait	*h* ^2^	SE	*P-value*	*m* ^2^	SE	*P-value*
PL	0.71	0.17	<0.01	0.20	0.12	0.02
IMF	0.89	0.16	<0.01	0.26	0.12	0.02

h^2^, heritability; m^2^, microbiability. The significant P-value bounds are P < 0.05.

### The genera that are significant association with percentage of leaf fat and intramuscular fat content

**FIGURE 2:**
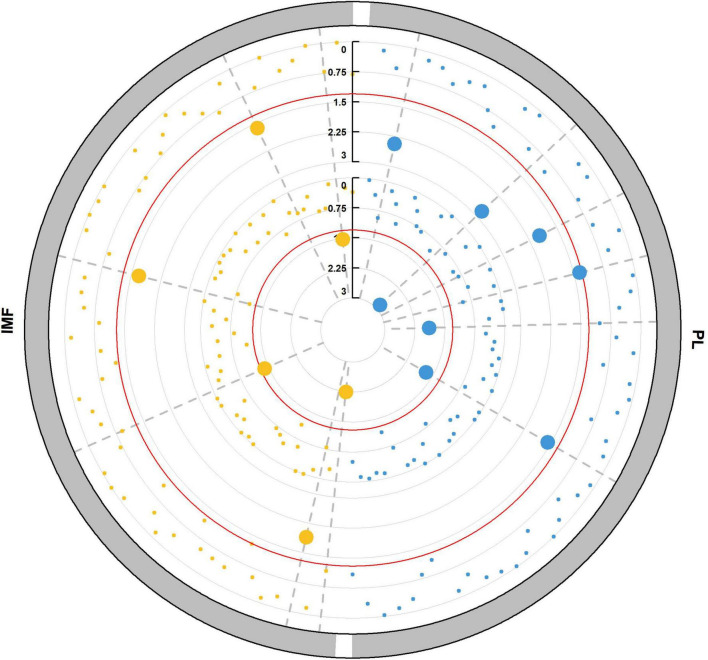
Microbial detections *via* Wilcoxon rank-sum tests between divergent groups. Microbial abundance in the highest 20% group with lowest 20% group traits. Displayed from the outer to the inner circle is the trait, the significance test in trait between the two groups with the highest and lowest microbial abundance (*P_*m*–trait_*), the significance test in each microbial abundance between the highest and lowest trait (*P_*trait*–*m*_*), where *p-values* are plotted as –log10 (*p-value*); the red line shows the significance threshold (*P* < 0.05). Each point represents a microbe, and the big point indicates the *p*-value passed the significance threshold. The dot on the same gray dashed line indicates that the *P*_*m–trait*_ and *P_*trait*–*m*_* values for one microbe are all <0.05.

To identify the candidate microbiome from the resource population, we tested the significance in traits or relative abundance between the highest 20% and lowest 20% microbial abundance or trait groups by Wilcoxon rank-sum test. We chose the microbes with significant difference in trait and genus relative abundance between divergent groups as candidate microbes (Figure 2) in the subsequently association analysis. PL and IMF had different candidate microbes. *Campylobacter* and *Paraprevotella* exhibited significant difference between divergent groups of PL, while PL also showed significant difference between divergent groups of *Campylobacter* and *Paraprevotella* (*P* < 0.05, Table 4). PL was 6.18% in the highest 20% group of *Campylobacter* relative abundance, while PL was 7.08% in the lowest 20% group (*P* < 0.05, Table 4). Then, PL was 6.25% in the highest 20% group of *Paraprevotella* relative abundance, while it was 6.95% in the lowest 20% group (*P* < 0.05, Table 4). For divergent Wilcoxon rank-sum test of IMF, *Actinobacillus*, *Dialister*, and *YRC22* exhibited significant differences in relative abundance between highest 20% and lowest 20% groups (*P* < 0.05, Table 4). For divergent Wilcoxon rank-sum test of genus relative abundance, *Anaeroplasma*, *Megasphaera*, and *Succinivibrio* exhibited significant differences between highest 20% and lowest 20% IMF (*P* < 0.05, Table 4). However, there were no overlapping genera between divergent groups of traits and genus relative abundance (Table 4).

**TABLE 4:** Microbiome relative abundance and trait Wilcoxon test of highest 20% and lowest 20% groups in the resource population.

Trait	Micro	The 20% pigs of		The 20% pigs of		*P-value*	The 20% pigs of		The 20% pigs of		*P-value*
		highest trait		lowest trait			highest micro		lowest micro		
		Micro	Micro	Micro	Micro		Trait	Trait	Trait	Trait	
		mean	SD	mean	SD		mean	SD	mean	SD	
PL	*Anaeroplasma*	0.04	0.05	0.03	0.04	0.12	7.01	1.12	6.28	1.08	<0.01
	***Campylobacter***	0.31	0.81	0.65	0.9	<0.01	6.18	1.2	7.08	1.29	<0.01
	*Desulfovibrio*	0.44	1.03	0.39	0.52	0.15	6.49	1.17	7.14	1.42	0.01
	*Escherichia*	0.91	2.13	1.71	4.82	0.11	6.3	1.25	6.79	1.28	0.04
	*Haemophilus*	0.01	0.02	0.03	0.08	0.01	6.23	1.12	6.68	1.64	0.09
	***Paraprevotella***	0.03	0.06	0.08	0.23	0.02	6.25	1.36	6.95	1.39	0.03
IMF	*Actinobacillus*	0.05	0.09	0.05	0.18	0.01	2.09	0.84	1.82	0.7	0.07
	*Anaeroplasma*	0.04	0.04	0.03	0.04	0.16	2.22	0.84	1.83	0.51	0.01
	*Dialister*	0.04	0.11	0.03	0.1	0.04	2.24	0.82	2	0.54	0.12
	*Megasphaera*	0.43	1.72	0.11	0.3	0.07	2.22	0.78	1.91	0.51	0.02
	*Succinivibrio*	0.01	0.02	0.04	0.09	0.27	1.78	0.66	2.07	0.65	0.02
	*YRC22*	1.01	1.02	0.65	0.64	0.03	2.08	0.8	1.89	0.61	0.2

PL, percentage of leaf fat; IMF, intramuscular fat content; SD, standard deviation. The significant P-value bounds are P < 0.05.

### Microbiome-wide association analysis of percentage of leaf fat and intramuscular fat content

The MWAS was used to investigate whether there were significant associations between genus relative abundance and traits (PL and IMF). The MWAS results are shown in Supplementary Table 5. The number of microbes significantly associated PL and IMF was 4 and 2, respectively (*P* < 0.05, Table 5). Notably, some genera showed high significant associations with traits. For example, *Anaeroplasma* was significantly associated with PL (*P* < 0.05), whereas *Sharpea* with IMF. Moreover, *Paraprevotella*, *Pasteurella*, and *Streptococcus* were also significantly associated with PL (*P* < 0.05), while *Helicobacter* exhibited significant association with IMF (*P* < 0.05).

**TABLE 5:** Microbes significantly associated with PL and IMF.

Trait	Genus	Beta	*P-value*
PL	*Anaeroplasma*	3.16	0.03
	*Paraprevotella*	−0.83	0.03
	*Pasteurella*	−1.01	0.04
	*Streptococcus*	0.04	0.03
IMF	*Helicobacter*	0.12	0.01
	*Sharpea*	2.53	<0.01

PL, percentage of leaf fat; IMF, intramuscular fat content. The significant P-value bounds are P < 0.05.

We adopted two methods (divergent Wilcoxon rank-sum test and MWAS) to screen microbes affecting PL and IMF at the genus level and found that *Paraprevotella* was significantly associated with PL in both methods (*P* < 0.05). As a significant candidate genus, *Paraprevotella* is one of the most important butyric acid-producing bacteria, and it may affect visceral fat deposition by producing butyric acid (a short-chain fatty acid).

## Discussion

Fat deposition trait is closely related to carcass and meat quality traits in pigs, and it affects economic income and feed consumption. Pig fat deposition traits are influenced by host genetics and gut microbiome. Our study found that PL and IMF were influenced by the interactions between host genetics and gut microbiome. Several studies have identified that host genetics have interacted with gut microbiota and some host genetic variations affect the gut microbiota (Li et al., 2019; Bergamaschi et al., 2020; Wen et al., 2021). In our study, the IMF showed 0.29 effect of interactions between host genetics and gut microbiome, but no independent effect of gut microbiome. The reason that the IMF showed no independent effect of gut microbiome may be that gut microbiota affects IMF by interacting with host genetics, but not alone. Then, we estimated the heritability and microbiability of PL and IMF. In this study, a total of 14,139,625 SNPs were obtained from whole-genome sequencing of 239 samples from the resource population. GCTA was used to construct the GRM and estimate heritability of PL and IMF. The heritability of PL and IMF was estimated as 0.71 and 0.89, respectively. The proportion of SNP variance has been reported to be SNP heritability of traits (Yang et al., 2017). Heritability is calculated by genome sequencing data to analyze the effect of genetics on human body weight (Yang et al., 2010). The heritability is influenced by breed (Lopez et al., 2018), sample size (Tang et al., 2020), and sequencing methods (Uemoto et al., 2017). In addition, the heritability depends on the population, because both the variation in additive and non-additive genetic factors of each trait, and the environmental variance are population-specific (Visscher et al., 2008). The study results showed that when environmental factors of the resource population were consistent, most of the variation that is observed in the present population is caused by variation in genotypes. Besides, PL and IMF of this resource population were not strongly selected, and their genetic variation was large. The pedigree information (Costa et al., 2015) and chip data (Won et al., 2018; Wurtz et al., 2018) were widely applied for quantitative trait locus (QTL) mapping. So far, no report on more than 10 million SNP markers used for estimating the heritability of quantitative traits in livestock and poultry is available. This study provides a reference for heritability estimation based on genome-wide SNP data.

In our study, heritability estimation model was consistent with microbiability estimation model. Both sex and BW were used as covariates to explore the influence of cecal microbiome on traits. To correct the effect of host genome on traits, the first five PCs generated by the GRM and the first two PCs generated at SNPs which were significantly associated with PL and IMF are considered as covariates. In the previous study, the first two PCs and the first five PCs of population structure were used as covariates to estimate microbiability (Wen et al., 2019b). In our study, the host heredity had major effect on traits. Several studies have reported that the microbiability of traits was lower than the heritability of traits in pigs (Camarinha-Silva et al., 2017; Tang et al., 2020; Khanal et al., 2021), chicken (Wen et al., 2019b), and cattle (Difford et al., 2018). In previous studies, the microbiability of the IMF was estimated as 0.13 in Enshi pigs (Tang et al., 2020), estimated as 0.03–0.06 following different models in commercial F1 sows composed of Yorkshire × Landrace or Landrace × Yorkshire (Khanal et al., 2021). The microbiability of IMF in the resource population was estimated to be 0.26, which was higher than that in the previous study. The reason for the difference in these studies’ results is that estimation of microbiability is affected by breed, environment, sample size, sequencing method, and estimation algorithm (Wen et al., 2019a). So far, few studies have described the microbiability of fat deposition traits in pigs, and estimation of microbiability of fat deposition traits in pigs needs to be further studied. Our estimated microbiability suggested that the gut microbiome had an influence on PL and IMF and provided a reference for microbiability estimation.

Pig fat deposition traits are affected by a large number of complex microbes and their metabolites which are closely related to host immune diseases, nutritional metabolism, and body behavior (Patil et al., 2020). To select microbes affecting traits, divergent Wilcoxon rank-sum test and MWAS were applied in our study. The divergent Wilcoxon rank-sum test has been used to analyze microbes in previous studies (Yang et al., 2016; Gardiner et al., 2020). MWAS has been proposed with reference to genome-wide association analysis to establish the relationship between traits and gut microbes, and it has been applied in basic research (Gilbert et al., 2016; Vollmar et al., 2020). In our study, the divergent Wilcoxon rank-sum test showed that *Campylobacter* and *Paraprevotella* were significantly associated with PL, and the MWAS results showed that *Anaeroplasma*, *Paraprevotella*, *Pasteurella*, and *Streptococcus* were significantly associated with PL. Among these genera, *Streptococcus* is reported as one of the most abundant bacteria in the gut microbes of Jinhua Pigs (Yang et al., 2018). Jinhua pig is an obese-type breed characterized by higher levels of intramuscular fat (Miao et al., 2009). MWAS results indicated that *Helicobacter* and *Sharpea* were significantly associated with IMF. *Sharpea azabuensis* has been reported to affect methane emissions in rumen, and it was viewed as one of the important driving factors of lactic acid production and utilization (Kamke et al., 2016). In previous studies, lactic acid can reduce fat synthesis and accumulation (Yonejima et al., 2013; Gan et al., 2020).

Divergent Wilcoxon rank-sum test and MWAS results indicated that *Paraprevotella* was significantly associated with PL, suggesting the influence of single genus on host phenotype. *Paraprevotella* belongs to *Bacteroidetes* phylum which could degrade cellulose and produce butyric acid (Gao et al., 2018; Hasan et al., 2018). Butyric acid is a short-chain fatty acid (SCFA), and SCFAs are closely related to glucose and lipid metabolism and energy metabolism (Dabke et al., 2019). It has been demonstrated that SCFAs enhance adipocyte differentiation in porcine adipose tissue (Li et al., 2014). The content of butyric acid has been reported to be positively correlated with the relative abundance of *Paraprevotella* in mouse feces (Fei et al., 2019). Butyric acid is a major energy source for intestinal epithelial cells and plays key functional roles in maintaining intestinal homeostasis and in overall health status. It can promote the development of the intestine and has antioxidant and anti-inflammatory effects (Fu et al., 2019). Our data revealed a significant negative correlation between the relative abundance of *Paraprevotella* and PL. *Paraprevotella* may affect the intestinal homeostasis by influencing the anabolism of butyric acid, thereby affecting the visceral fat deposition in pigs, but the mechanism of *Paraprevotella* on affecting fat deposition in pig needs to be further studied.

## Conclusion

This study revealed that PL and IMF were influenced by the host genetics–gut microbiome interaction. Through divergent Wilcoxon rank-sum test and microbiome-wide association analysis, we screened out *Paraprevotella* which were significantly associated with PL. Overall, this study reveals the effect of host genetics and gut microbiome on pig fat deposition traits and provides a reference for the genetic improvement of pig fat deposition traits, which can affect pig fat deposition by altering the gut microbiome.

## Data availability statement

The genotyping data in PLINK binary format is available from the Figshare database (https://figshare.com/s/c0ece21cf32f35833569). The 16S ribosomal RNA sequencing data are available in the Sequence Read Archive (SRA) repository (SRA accession number PRJNA868520). Further information on the original data can be directed to the corresponding author.

## Ethics statement

This animal study was reviewed and approved by the Animal Care and Use Committee of Huazhong Agricultural University.

## Author contributions

YW collected the samples, performed the data analysis and interpretation, and wrote the manuscript. PZ performed the data analysis and wrote the manuscript. MF, ZL, and TW measured pig phenotype and collected samples. ZW and XL participated in constructing mathematical models. BL and XZ conceived and designed the study and manuscript revision. All authors contributed to manuscript revision, read, and approved the submitted version.

## Footnotes

1
https://www.hiblup.com/

2
https://cran.r-project.org/web/packages/gaston

## Supplementary material

Supplementary Figure 1 Distribution of fat deposition traits. **(A–H)** Distribution of backfat thickness at shoulder (SBFT), backfat thickness at loin (LBFT), backfat thickness at rump (RBFT), average backfat thickness (ABFT), percentage of leaf fat (PL), percentage of caul fat (PC), percentage of leaf fat, and caul (PLC), and intramuscular fat content (IMF).
