# Supplementary figures and images for: Effect of host genetics and gut microbiome on fat deposition traits in pigs
Source: Front Microbiol. 2022 Sep 20;13:925200. doi: 10.3389/fmicb.2022.925200 (PMC9530793; doi:10.3389/fmicb.2022.925200)

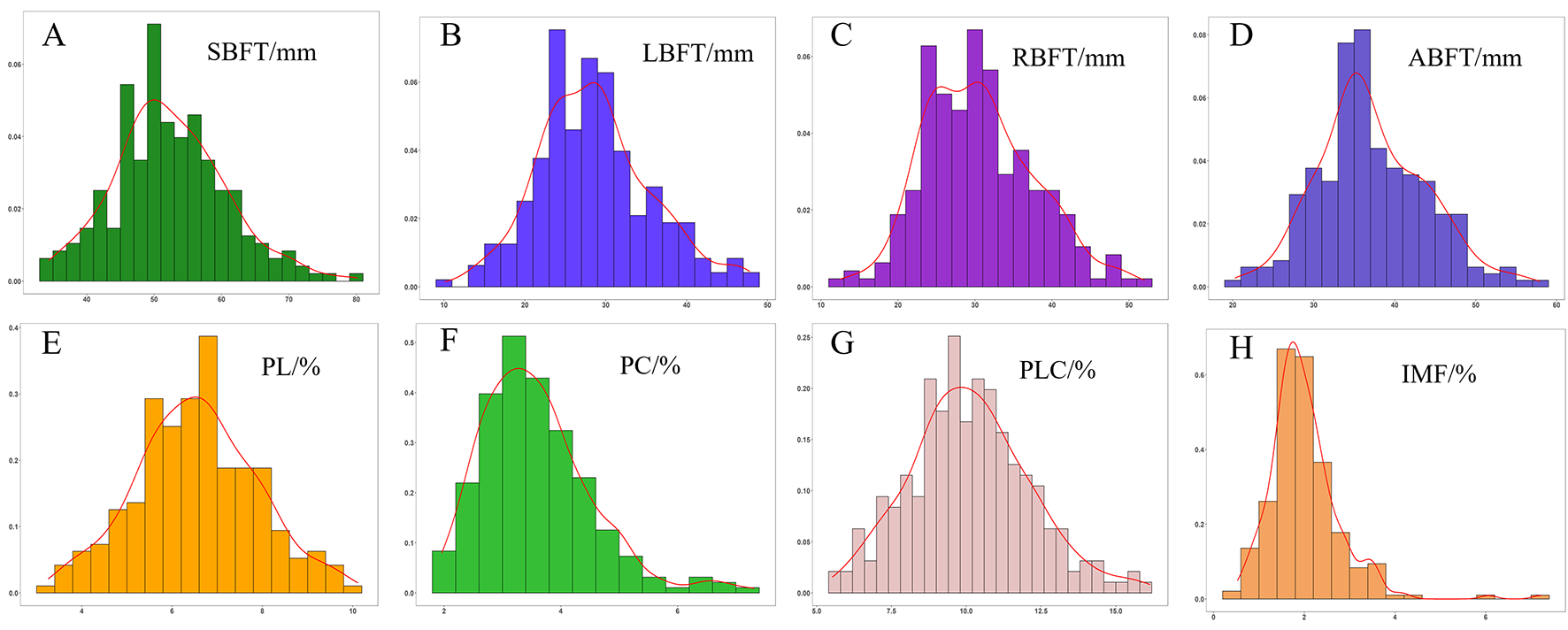

Supplement: Supplementary Figure 1 — Distribution of fat deposition traits. (A–H) Distribution of backfat thickness at shoulder (SBFT), backfat thickness at loin (LBFT), backfat thickness at rump (RBFT), average backfat thickness (ABFT), percentage of leaf fat (PL), percentage of caul fat (PC), percentage of leaf fat, and caul (PLC), and intramuscular fat content (IMF). [file Image_1.TIF]
